# MB-MSTFNet: A Multi-Band Spatio-Temporal Attention Network for EEG Sensor-Based Emotion Recognition

**DOI:** 10.3390/s25154819

**Published:** 2025-08-05

**Authors:** Cheng Fang, Sitong Liu, Bing Gao

**Affiliations:** 1 Key Laboratory of Civil Aviation Thermal Hazards Prevention and Emergency Response, Civil Aviation University of China, Tianjin 300300, China; fangcheng0513@163.com; 2 College of Electronic Information and Automation, Civil Aviation University of China, Tianjin 300300, China; liusitong001017@163.com; 3 Engineering Techniques Training Center, Civil Aviation University of China, Tianjin 300300, China

**Keywords:** electroencephalograph (EEG), convolutional neural network (CNN), bidirectional gated recurrent unit (BiGRU), multi-head attention (MHA), emotion signal recognition, Inception module

## Abstract

Emotion analysis based on electroencephalogram (EEG) sensors is pivotal for human–machine interaction yet faces key challenges in spatio-temporal feature fusion and cross-band and brain-region integration from multi-channel sensor-derived signals. This paper proposes MB-MSTFNet, a novel framework for EEG emotion recognition. The model constructs a 3D tensor to encode band–space–time correlations of sensor data, explicitly modeling frequency-domain dynamics and spatial distributions of EEG sensors across brain regions. A multi-scale CNN-Inception module extracts hierarchical spatial features via diverse convolutional kernels and pooling operations, capturing localized sensor activations and global brain network interactions. Bi-directional GRUs (BiGRUs) model temporal dependencies in sensor time-series, adept at capturing long-range dynamic patterns. Multi-head self-attention highlights critical time windows and brain regions by assigning adaptive weights to relevant sensor channels, suppressing noise from non-contributory electrodes. Experiments on the DEAP dataset, containing multi-channel EEG sensor recordings, show that MB-MSTFNet achieves 96.80 ± 0.92% valence accuracy, 98.02 ± 0.76% arousal accuracy for binary classification tasks, and 92.85 ± 1.45% accuracy for four-class classification. Ablation studies validate that feature fusion, bidirectional temporal modeling, and multi-scale mechanisms significantly enhance performance by improving feature complementarity. This sensor-driven framework advances affective computing by integrating spatio-temporal dynamics and multi-band interactions of EEG sensor signals, enabling efficient real-time emotion recognition.

## 1. Introduction

Emotion, as a critical indicator of human psychological and physiological states, comprehensively reflects the interaction among sensory experiences, cognition, and behavior. In artificial intelligence, emotion recognition technologies achieve precise emotional state perception by fusing multi-modal information such as facial micro-expressions, body language, and speech semantics [1,2]. Among these, video-based emotion analysis—leveraging facial expressions, gesture dynamics, and scene context—has accumulated extensive research. For instance, methods based on convolutional neural networks (CNNs) and transformers have achieved remarkable performance in recognizing emotional states from video footage: a unified transformer-based multimodal framework for facial expression analysis integrates CNN-extracted visual features with transformer-based sequence modeling, effectively capturing micro-expression sequences and subtle posture changes [3]; meanwhile, studies employing transformer-based interactive multi-modal attention networks have demonstrated superior performance in video emotion recognition tasks, highlighting the ability to dynamically fuse visual, auditory, and textual cues across continuous video frames while modeling temporal–spatial dependencies [4]. This body of research not only highlights the pivotal role of multi-modal fusion in advancing emotion recognition but also provides critical methodological insights—specifically, the proven value of integrating heterogeneous signals to boost robustness—directly informing our approach to incorporating EEG data as a complementary modality. Unlike visual or audio signals that primarily reflect emotional expressions, EEG captures the neural correlates of emotional experiences at their source, offering unique insights into underlying psychological processes; recent advancements in machine learning, signal processing techniques, and EEG acquisition devices [5,6,7] have thus propelled EEG-based emotion recognition to the forefront of interdisciplinary research at the intersection of artificial intelligence and biomedicine. As a direct recording of brain electrical activity, EEG signals offer high temporal resolution and real-time monitoring capabilities, providing a novel perspective for emotion recognition and enabling technological innovations in intelligent interaction and mental health diagnosis [8,9].

Feature extraction constitutes a core component of EEG emotion recognition research. Early traditional methods relied on manually designed features, with algorithms such as support vector machines (SVM) [10], random forests (RF) [11], and k-nearest neighbors (KNN) demonstrating limited performance due to their inability to extract deep emotional features, resulting in low recognition accuracy. Duan et al. [12] verified the sensitivity of differential entropy (DE) to emotional states, and Alsolamy et al. [13] found that analyzing the power spectral density (PSD) of EEG signals with an SVM classifier can effectively distinguish human emotional states. However, these methods are limited to single frequency-domain feature analysis, neglecting the complementarity of feature fusion and the potential of spatio-temporal dynamic coupling.

The advent of deep learning has driven a paradigm shift in EEG emotion recognition, leveraging data-driven hierarchical feature learning as its core advantage. In spatial modeling, early efforts focused on extracting local electrode correlations: Hao et al. [14] proposed a spatial modeling method that constructs a 3D matrix based on the rearrangement of EEG time-domain features, utilizing a CNN with univariate and multivariate convolutional layers to exploit intra-channel and inter-channel features and their correlations. As research advanced, spatiotemporal hybrid models emerged to bridge spatial details and temporal dynamics: Chakravarthi et al. [15] proposed a CNN-LSTM framework that uses convolutional neural networks to extract local spatial features from electrode configurations and integrates long short-term memory (LSTM) for temporal dynamics modeling, enabling hierarchical spatiotemporal feature learning. They further enhanced performance by fusing homogeneous CNN and LSTM classifiers with the ResNet152 architecture, demonstrating the value of complementary model fusion. Graph neural networks (GNNs) later refined spatial modeling by explicitly capturing topological relationships: Song et al. [16] developed a dynamic graph convolutional neural network (DGCNN) that dynamically learns channel relationships via adjacency matrices during training to extract discriminative features. Feng et al. [17] proposed a spatiotemporal graph convolutional long short-term memory network (ST-GCLSTM), integrating spatial graph convolutions with attention-enhanced temporal LSTM to dynamically model spatiotemporal correlations in EEG signals.

In temporal modeling and multi-band collaboration, Yang et al. [18] introduced the EEG-TCN model, which uses causal and dilated convolutions to expand temporal receptive fields and capture long-range temporal dependencies. Tang et al. [19] proposed the MD^2^GRL framework, integrating GRU with time-frequency features and dynamically adjusting graph structures using band power to enable cross-band information interaction. Yi et al. [20] developed a Transformer Capsule Network (TC-Net). It employs Transformer blocks to model global temporal dependencies across non-overlapping time windows and introduces an EEG-PatchMerging strategy to extract hierarchical local features. This design enhances multi-dimensional fusion of temporal, spectral, and spatial information, improving emotion recognition performance. Miao et al. [21] proposed a Multiple Frequency Band Parallel Spatial-Temporal 3D Deep Residual Learning Framework (MFBPST-3D-DRLF), which enhances temporal dependency modeling and cross-band collaboration by processing multi-band EEG features in parallel and integrating spatiotemporal information.

Notwithstanding these advancements, critical limitations persist: For instance, while models like CNN-LSTM [15] integrate spatial and temporal features, they rely on shallow concatenation rather than deep semantic fusion. Methods such as MD^2^GRL [19] and MFBPST-3D-DRLF [21], despite addressing cross-band collaboration, still use simple band-wise concatenation that overlooks hierarchical correlations between frequency bands. Additionally, dynamic modeling in EEG-TCN [18] and TC-Net [20] lacks robustness to time-varying signals, and cross-brain-region feature interactions remain under-explored in most frameworks.

To address these limitations, this paper presents MB-MSTFNet, an EEG emotion recognition model adapted to emotional feature learning under the valence–arousal framework. The model constructs fused 3D feature tensors of DE and PSD to explicitly encode frequency–space–time correlations. It incorporates a multi-scale CNN-Inception and BiGRU cascaded architecture for hierarchical spatial feature extraction and bidirectional temporal modeling, and integrates a multi-head self-attention mechanism to focus on critical time windows and brain regions—suppressing noise and enhancing discriminative dynamic feature representation [22]. Experimental validations on the DEAP dataset demonstrate the model’s effectiveness, achieving high emotion recognition accuracy and robust performance across different emotional states.

## 2. Proposed Method

To address the research objectives, the present study utilizes the DEAP dataset [23,24,25], which was collected by institutions including Queen Mary University of London. This dataset comprises EEG and peripheral physiological signals recorded from 32 participants while they viewed 40 one-minute music video excerpts. The experiment employed a 32-channel EEG electrode cap based on the international 10–20 standard lead system for synchronous signal acquisition. Each experimental trial lasted 63 s, consisting of a 3 s resting baseline period (during which participants remained silent) followed by a 60 s emotion induction phase with synchronous audio-visual stimulation. Post-recording, participants completed the SAM emotional self-rating scale, providing subjective scores (ranging from 1 to 9) across four dimensions: valence, arousal, dominance, and liking. The emotion recognition task in this study is based on the circumplex model of emotion [26], which divides emotions into two core dimensions: valence (ranging from negative to positive) and arousal (ranging from low to high). The emotion labels in the DEAP dataset are defined by participants’ subjective ratings for valence and arousal, where scores ≥ 5 are classified as “high” and scores < 5 as “low”. This classification forms four emotion types—HVHA (high valence–high arousal), HVLA (high valence–low arousal), LVHA (low valence–high arousal), and LVLA (low valence–low arousal)—which directly correspond to the two-dimensional structure of the circumplex model.

**Figure 1:**
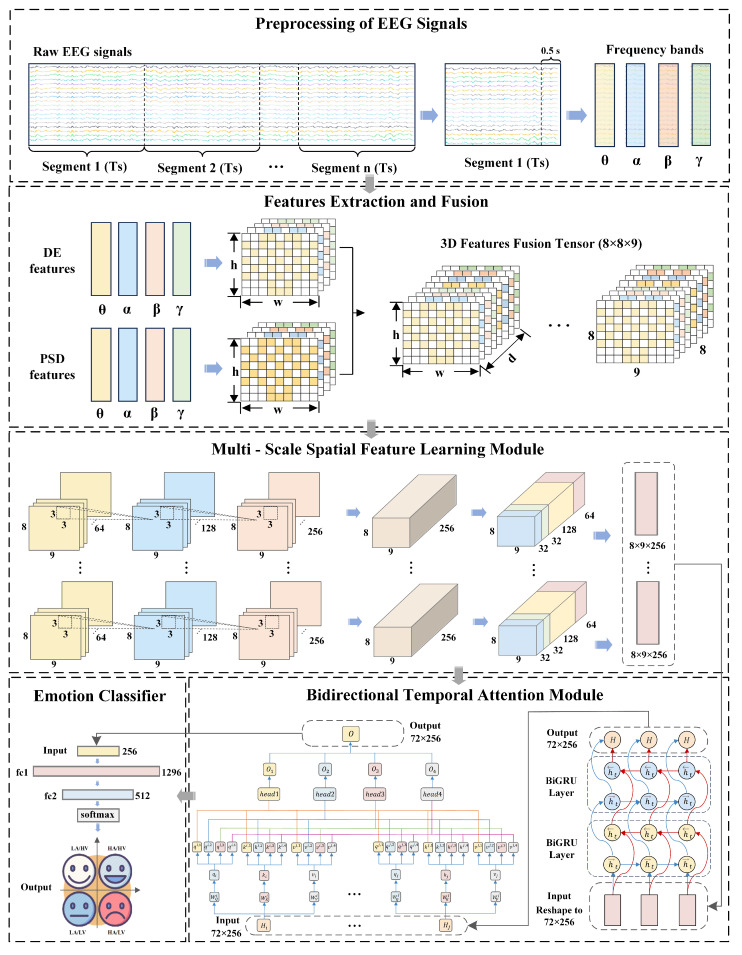
The framework of the proposed EEG-based emotion recognition model MB-MSTFNet.

The architecture of the proposed MB-MSTFNet emotion recognition model is depicted in Figure 1.

The model comprises the following components:

(1) Decompose raw EEG signals into distinct frequency bands corresponding to different brain states and partition them into temporal segments.
(2) Compute DE and PSD for each frequency band slice, map these features onto the brain’s spatial topology, and concatenate them to form 3D fused features.
(3) Extract local EEG features using a CNN and perform feature fusion via multi-scale convolution and pooling in the Inception module.
(4) After dimensionality reduction via a max pooling layer, flatten the features and use BiGRU layers to capture bidirectional temporal dependencies in the feature sequence, revealing the signals’ dynamic patterns.
(5) Integrate a multi-head attention mechanism to calculate weights across different subspaces, enabling adaptive focusing on critical features and suppression of interfering information.
(6) Map the processed features to a four-class classification space through a fully connected layer, using the softmax function as the classifier to generate recognition outputs.

### 2.1. Three-Dimensional Feature Representation via DE and PSD Fusion

Based on the DEAP dataset characteristics, the EEG signal preprocessing and feature extraction pipeline is structured as follows: Initially, raw EEG signals from the DEAP dataset are decomposed into four distinct frequency bands using a Butterworth filter, with each band corresponding to canonical neural oscillations: theta (θ, 4–8 Hz), alpha (α, 8–13 Hz), beta (β, 13–30 Hz), and gamma (γ, 30–45 Hz). Given the temporal resolution of the dataset (128 Hz sampling rate), non-overlapping signal fragments were created using a 0.5 s sliding window (64 samples per fragment), effectively dividing the 60 s emotion-inducing period into 120 periods of shorter duration. No resampling was performed in this study to maintain the original temporal resolution of the signal, ensuring that the dynamic properties of EEG oscillations were preserved for subsequent feature extraction. Baseline correction is then applied to each segment, utilizing the 3 s resting baseline data from the dataset to mitigate baseline drifts.

For each frequency band, the DE and PSD features are selected for extraction and fusion. Given an EEG segment of a specific length that adheres to a Gaussian distribution $N(\mu ,\sigma_{i}^{2})$, the DE is calculated as follows:

(1) $$DE\left(X\right)=-\int_{-\infty }^{\infty }\frac{1}{\sqrt{2\pi \sigma_{i}^{2}}}e^{-\frac{{\left(x-\mu \right)}^{2}}{2\sigma_{i}^{2}}}\log\left(\frac{1}{\sqrt{2\pi \sigma_{i}^{2}}}e^{-\frac{{\left(x-\mu \right)}^{2}}{2\sigma_{i}^{2}}}\right)dx$$

The DE of EEG signals in the *i*-th frequency band can be expressed as [27,28,29]:

(2) $$DE\left(X\right)=h_{i}\left(X\right)=\frac{1}{2}\log\left(2\pi e\sigma_{i}^{2}\right)$$

Herein, $h_{i}$ denotes the DE of the EEG signal in the *i*-th frequency band, and $\sigma_{i}^{2}$ represents the signal variance.

The PSD features are computed using Welch’s periodogram method [30]. First, the signals are preprocessed with average reference correction [31] to suppress common-mode noise:

(3) $$V_{i}^{\mathrm{new}}=V_{i}-\frac{1}{32}\sum_{j=1}^{32}V_{j}$$

where 32 corresponds to the number of EEG electrodes, $V_{i}$ is the raw EEG signal from the *i*-th electrode (i=1,2,…,32), $V_{j}$ is the raw EEG signal from the *j*-th electrode (a summation variable, j=1,2,…,32), and $V_{i}^{\mathrm{new}}$ denotes the corrected signal of the *i*-th electrode after average reference correction.

Next, the preprocessed signals are segmented into non-overlapping windows of 0.5s length. Given a sampling rate of $f_{s}=128\mathrm{Hz}$, each window contains N=64 samples. A Hanning window is applied to minimize spectral leakage:

(4) $$w\left(n\right)=0.5\left(1-\cos\left(\frac{2\pi n}{N-1}\right)\right),\;n=0,1,\ldots ,N-1$$

where w(n) is the Hanning window function, *n* is the sample index within the window (ranging from 0 to N−1), and *N* denotes the number of samples per window.

An FFT of size $N_{\mathrm{FFT}}=128$ is applied to each windowed segment. For non-overlapping windows, Welch’s method simplifies to a single-segment estimation (K=1):

(5) $$PSD\left(f\right)=\frac{1}{K\cdot N_{\mathrm{FFT}}}\sum_{k=1}^{K}{\left|X_{k}\left(f\right)\right|}^{2}$$

where $X_{k}\left(f\right)$ is the FFT result of the *k*-th windowed segment, and $1/(K\cdot N_{\mathrm{FFT}})$ normalizes the PSD to account for the Hanning window’s energy.

Finally, the power in each frequency band is approximated by summing the PSD values over the corresponding frequency bins:

(6) $$P_{\mathrm{band}}\approx \sum_{f_{i}\in \mathrm{band}}PSD\left(f_{i}\right)\cdot \Delta f,\;\mathrm{with}\;\Delta f=\frac{f_{s}}{N_{\mathrm{FFT}}}=1,\mathrm{Hz}$$

where $P_{\mathrm{band}}$ denotes the power of a specific frequency band, $f_{i}$ is the center frequency of the *i*-th frequency bin within the band, and the frequency bands are defined as θ, α, β, and γ.

**Figure 2:**
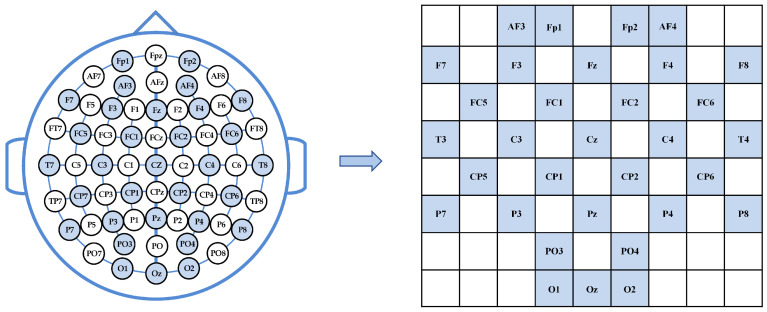
DEAP Dataset Electrode 2D Mapping.

To explore the correlations between the features of different frequency bands and time slices, spatial topology mapping is conducted based on the international 10–20 system [32] electrode layout. This process transforms one-dimensional vectors into two-dimensional matrices. Specifically, the 32-dimensional features of each frequency band are reconstructed into an 8 × 9 matrix to encode the spatial distribution characteristics of the EEG signals. In this study, the DEAP dataset is employed to validate the performance of the proposed model. In the DEAP dataset, 32 electrode points are marked with blue circles, as illustrated in Figure 2.

For the feature vector $v_{k}\in R^{32}$ of the *k*-th frequency band, a two-dimensional matrix $M(v_{k})$ is generated through the mapping function L, which satisfies

(7) $$M\left(v_{k}\right)\left[i,j\right]=\left\{\begin{array}{cc} v_{m} & \mathrm{if}(i,j)\in L(m)\\ 0 & \mathrm{otherwise} \end{array}\right.$$

where $v_{k}$ is the 32-dimensional feature vector of the *k*-th frequency band, $M(v_{k})$ is the mapped 2D matrix, L(m) is the mapping function that links the *m*-th element of $v_{k}$ to matrix position (i,j), $v_{m}$ is the *m*-th element of $v_{k}$, and (i,j) denotes the row and column indices of the matrix.

To preserve the frequency-band-specific information, we stack the two-dimensional matrices of the four frequency bands along the frequency-band dimension to construct a three-dimensional tensor $T\in R^{4\times 8\times 9}$, expressed as

(8) $$T=[M_{\theta },M_{\alpha },M_{\beta },M_{\gamma }]$$

where *T* is the 3D tensor, $M_{\theta }$, $M_{\alpha }$, $M_{\beta }$, $M_{\gamma }$ are the 2D matrices corresponding to θ, α, β, γ bands, and 4×8×9 denotes dimensions: number of frequency bands, matrix rows, and matrix columns.

Following the international 10–20 system, we map the 32-channel DE values to an 8 × 9 grid:

(9) $$M_{\theta }=L\left(v_{\theta }\right),M_{\alpha }=L\left(v_{\alpha }\right),M_{\beta }=L\left(v_{\beta }\right),M_{\gamma }=L\left(v_{\gamma }\right)$$

where $M_{\theta }$, $M_{\alpha }$, $M_{\beta }$, $M_{\gamma }$ are the 2D matrices of DE features for each band, and *L* is the spatial mapping function.

Subsequently, we stack these matrices along the frequency-band dimension to generate the DE tensor $T_{\mathrm{DE}}\in R^{4\times 8\times 9}$, where $T_{\mathrm{DE}}$ denotes the 3D tensor of DE features, with four frequency bands, eight rows, and nine columns.

For PSD features, we first calculate the power spectral density of each band using Welch’s method to obtain a 128-dimensional feature vector. To ensure spatial alignment, we reuse the DE spatial mapping rule L to construct the PSD tensor $T_{\mathrm{PSD}}\in R^{4\times 8\times 9}$, where $T_{\mathrm{PSD}}$ is the 3D tensor of PSD features with the same spatial dimensions as $T_{\mathrm{DE}}$.

Finally, we fuse the DE and PSD features along the channel dimension to form a combined feature tensor. $T_{\mathrm{Fusion}}\in R^{8\times 8\times 9}$, defined as

(10) $$T_{\mathrm{Fusion}}\left[c,i,j\right]=\left\{\begin{array}{cc} T_{\mathrm{DE}}\left[c,i,j\right] & 0\leq c<4\\ T_{\mathrm{PSD}}\left[c-4,i,j\right] & 4\leq c<8 \end{array}\right.$$

where $T_{\mathrm{Fusion}}$ is the fused 3D tensor, *c* is the channel index (0–7), (i,j) are the row and column indices of the spatial grid, $T_{\mathrm{DE}}\left[c,i,j\right]$ denotes the DE feature at channel *c*, row *i*, column *j*, and $T_{\mathrm{PSD}}\left[c-4,i,j\right]$ denotes the PSD feature at adjusted channel c−4, row *i*, column *j*.

**Figure 3:**
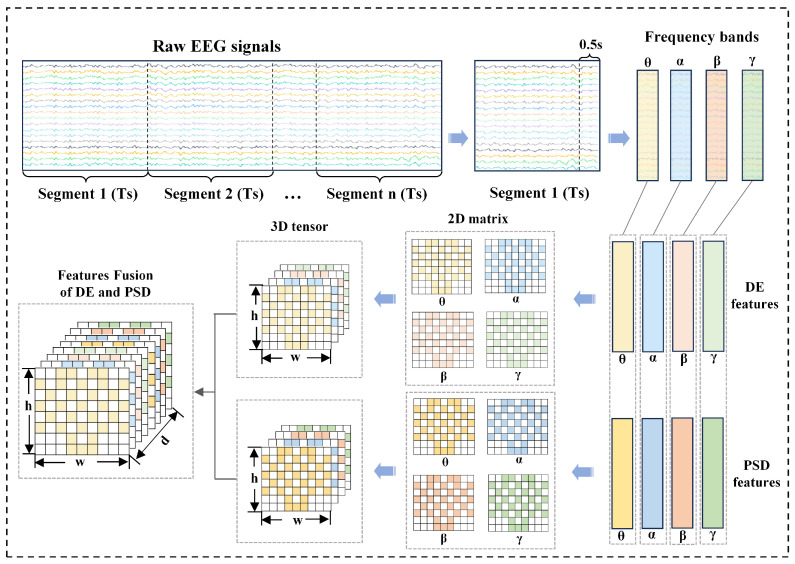
Three-dimensional tensor for EEG spatial-frequency representation.

This operation concatenates DE and PSD along the frequency-band dimension to create composite features, as illustrated in Figure 3.

### 2.2. Multi-Scale Spatial Feature Learning

**Figure 4:**
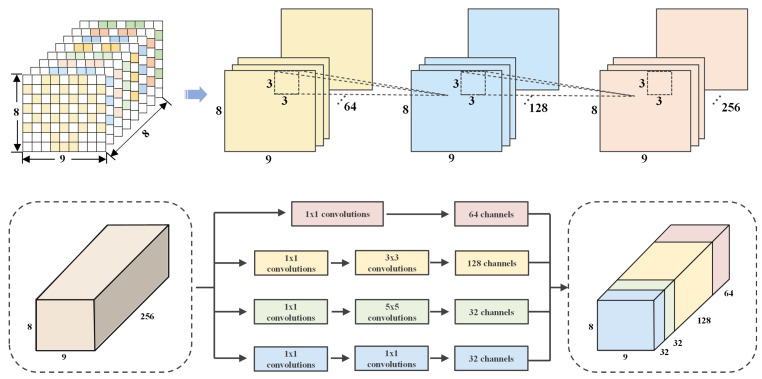
CNN-Inception for multi-scale spatial extraction.

To address the multi-scale rhythmic characteristics of EEG signals and the limitation of fixed receptive fields in traditional convolutional layers [33], this paper presents a cascaded CNN-Inception module incorporating convolution and multi-branch fusion, as depicted in Figure 4.

The proposed module comprises a hierarchical convolutional path and a multi-branch fusion path. The hierarchical path utilizes three 3 × 3 convolutional layers, each followed by a ReLU activation, to gradually expand feature dimensions. This enables the module to learn from low-level pixel-wise features to high-level abstract features. The multi-branch fusion path contains four parallel branches, designed to perform multi-scale feature extraction via different kernel sizes and pooling operations. These branches encompass a wide range of features, from point-wise details to large-region patterns. The outputs of each branch are concatenated along the channel dimension to ensure consistent feature dimensions. This design not only preserves the ability to extract fine-grained local features but also facilitates effective global pattern capture through multi-scale fusion, offering a more adaptable feature representation framework for EEG signal analysis.

By utilizing 3 × 3 convolutional kernels, the model captures spatial patterns within electrode neighborhoods. This approach accurately models the subtle correlations between adjacent electrodes, thereby refining local features with enhanced precision.

### 2.3. Bidirectional Temporal Attention Fusion

**Figure 5:**
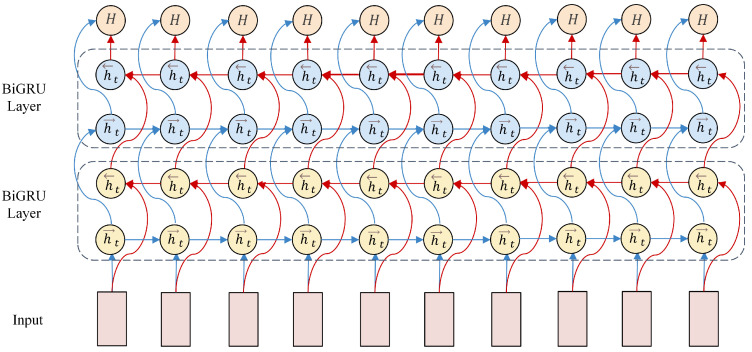
BiGRU Bidirectional Temporal Modeling.

To eliminate redundant information in EEG data after processing by convolutional and Inception modules, a 2 × 2 max-pooling layer is employed for dimensionality reduction. The pooled feature maps undergo two reshaping operations to meet the input format requirements of the BiGRU layer. For constructing the sequential input to the BiGRU, the original 60 s EEG signal is first segmented into consecutive 0.5 s epochs, and while preserving their temporal order, every eight consecutive epochs are grouped into a temporal sequence, corresponding to a 4 s time window. Each 0.5 s epoch is processed through a CNN-Inception module, which transforms it into a fixed-dimensional spatial feature vector. As a result, a sequence of eight epochs is represented as a matrix where one dimension denotes time steps and the other represents spatial features at each step. These matrices are fed into the BiGRU, in which the forward pass processes the sequence in chronological order to capture past-to-present dependencies, and the backward pass processes it in reverse to model future-to-current relationships. This bidirectional modeling enables the exploitation of long-range temporal dynamics within the 4 s window, thus aligning with the sequential processing requirements of recurrent neural networks. The reshaped input vectors integrate rich spatial feature information, facilitating sequence modeling by the BiGRU in the temporal dimension. In the BiGRU architecture, the hidden layer size is set to 128, with the output dimension of the hidden state vector at each time step matching this size, which determines the complexity of feature representation for sequential data. The BiGRU unit dynamically updates the 128-dimensional hidden state at each time step based on the current input and the previous hidden state, thereby effectively extracting temporal features from EEG signals. Bidirectional temporal modeling by the BiGRU is illustrated in Figure 5.

The model adopts a two-layer stacked BiGRU architecture. The first BiGRU layer carries out initial processing on the input data to extract basic temporal features, whose output serves as the input to the second BiGRU layer. The second BiGRU layer further learns more complex feature combinations and long-term dependencies based on the first-layer’s output.

The BiGRU consists of a forward GRU and a backward GRU. The forward GRU processes input data in chronological order to capture information before the current time step, while the backward GRU processes data in reverse order to capture information after the current time step.

For the GRU unit, the formulas for the update gate $z_{t}$, reset gate $r_{t}$, candidate hidden state ${\tilde{h}}_{t}$ and hidden state $h_{t}$ are as follows [34]:

(11) $$z_{t}=\sigma \left(W_{zh}x_{t}+U_{zh}h_{t-1}+b_{z}\right)$$

(12) $$r_{t}=\sigma \left(W_{rh}x_{t}+U_{rh}h_{t-1}+b_{r}\right)$$

(13) $${\tilde{h}}_{t}=\tanh\left(W_{hh}x_{t}+r_{t}⊙\left(U_{hh}h_{t-1}\right)+b_{h}\right)$$

(14) $$h_{t}=\left(1-z_{t}\right)⊙h_{t-1}+z_{t}⊙{\tilde{h}}_{t}$$

where $z_{t}$ and $r_{t}$ are the update gate and reset gate at time *t*, respectively, sigmoid-activated with output range [0,1]; ${\tilde{h}}_{t}$ is the candidate hidden state at time *t*, activated by the tanh function; $h_{t}$ denotes the hidden state at time *t*, which is fused from the previous hidden state $h_{t-1}$ and the candidate hidden state ${\tilde{h}}_{t}$ via the update gate $z_{t}$; $x_{t}$ is the input at time *t*, and $h_{t-1}$ is the hidden state at time t−1; $W_{zh},W_{rh},W_{hh}$ are weight matrices that map the input $x_{t}$ to the gates and hidden state, while $U_{zh},U_{rh},U_{hh}$ are weight matrices that map the previous hidden state $h_{t-1}$ to the gates and hidden state; $b_{z},b_{r},b_{h}$ represent bias terms; and ⊙ denotes the element-wise multiplication operation.

The forward GRU and backward GRU compute the forward hidden state ${\vec{h}}_{t}$ and backward hidden state ${\overset{\leftarrow }{h}}_{t}$, respectively. Finally, the outputs of the forward and backward GRUs are concatenated along the hidden state dimension:

(15) $$output=[{\vec{h}}_{t};{\overset{\leftarrow }{h}}_{t}]$$

The output *H* of the BiGRU serves as the input to the multi-head attention mechanism, in which query, key, and value all take *H* as their value (i.e., Q=K=V=H). Firstly, linear transformations are conducted on the *Q*, *K* and *V* to obtain $q_{i},k_{i}$ and $v_{i}$:

(16) $$q_{i}=QW_{Q}^{i}$$

(17) $$k_{i}=KW_{K}^{i}$$

(18) $$v_{i}=VW_{V}^{i}$$

Here, $W_{Q}^{i}$, $W_{K}^{i}$ and $W_{V}^{i}$ are the weight matrices for the *i*-th head (i=1,...,4). These linear transformations allow the model to learn feature representations in different subspaces, enhancing its expressive power.

Head-splitting operations are performed on $q_{i},k_{i}$ and $v_{i}$:

(19) $$q_{split}^{i}=q_{i}.view\left(B,-1,4,64\right).permute\left(0,2,1,3\right)$$

(20) $$k_{split}^{i}=k_{i}.view\left(B,-1,4,64\right).permute\left(0,2,1,3\right)$$

(21) $$v_{split}^{i}=v_{i}.view\left(B,-1,4,64\right).permute\left(0,2,1,3\right)$$

Here, *B* is the batch size, and the dimension of each head is 64. The head-splitting operation partitions the input feature vectors into multiple subspaces, allowing each head to focus on different feature representations.

Attention scores are computed as

(22) $$\mathrm{Attention}\left(q_{\mathrm{split}}^{i},k_{\mathrm{split}}^{i}\right)=\frac{q_{\mathrm{split}}^{i}{\left(k_{\mathrm{split}}^{i}\right)}^{T}}{\sqrt{d_{k}}}$$

where $\sqrt{d_{k}}$ is a scaling factor to prevent dot-product results from being too large, stabilizing the gradient.

The output for each head is calculated as

(23) $$O_{i}=A_{i}v_{split}^{i}$$

where $A_{i}$ is the *i*-th head’s attention weights (softmax-normalized); $O_{i}$ is its weighted value output.

**Figure 6:**
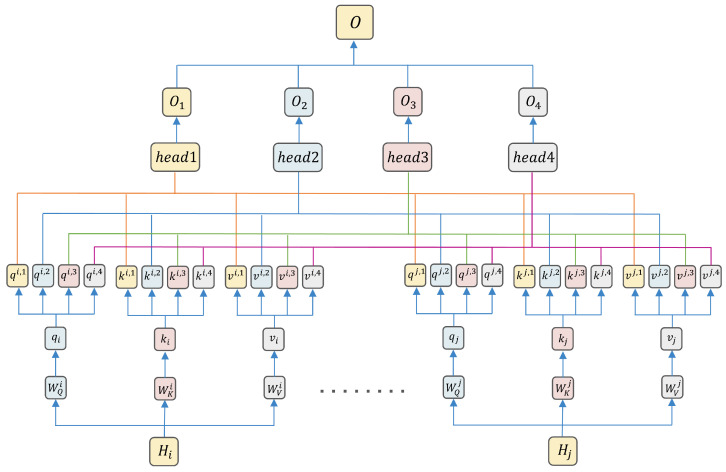
Multi-head attention for key emotional feature capture.

Concatenating the outputs of all heads and applying a linear transformation yields the final output:

(24) $$O=\mathrm{Concat}\left(O_{1},O_{2},O_{3},O_{4}\right)W_{o}$$

where $W_{o}$ is the weight matrix of the output layer. This concatenation and linear transformation fuse features learned by multiple heads to obtain the final feature representation. The capture of key emotional features by multi-head attention is shown in Figure 6.

The fusion of BiGRU and multi-head attention realizes complementary advantages, facilitating comprehensive capture of EEG-signal temporal features and precise focusing on key emotional features. This synergy significantly enhances the model’s ability to extract and represent emotional information from EEG signals, effectively improving feature quality and discriminability.

The input size, output size, and number of parameters for each layer of MB-MSTFNet on the DEAP dataset are listed in Table 1.

## 3. Experiments

In this section, we use the public EEG dataset DEAP to validate the effectiveness of the proposed MB-MSTFNet model for emotion recognition. We first elaborate on the experimental setup and then present the experimental results.

### 3.1. Experimental Setup

In the DEAP dataset, to preserve the spatial arrangement of electrodes, a 3D feature structure was employed. Each sample is organized into a matrix featuring spatial dimensions (h = 8, w = 9) alongside a temporal dimension. The segment length was set to 0.5 s, and each of the 40 videos per subject was split into 120 non-overlapping 0.5 s slices. Through structured aggregation, consecutive 0.5 s slices are grouped into temporal windows to form individual samples, ensuring the retention of temporal continuity while preserving electrode spatial arrangement. Consequently, for 32 subjects, the total number of 3D samples amounts to 153,600, with each sample structured as (8, 8, 9) to retain electrode spatial-frequency features.

For validation, five-fold cross-validation was carried out within each subject. For each subject, their 4800 samples (40 videos × 120 slices) were randomly partitioned into five folds. In each iteration, one fold served as test data, while the remaining four folds were combined for training. Subsequent to training on the combined training set, the model was evaluated on the held-out test fold. This process was repeated for all five folds per subject, and results such as accuracy were aggregated. Ultimately, the average accuracy across all folds and subjects, as well as the per-subject accuracy, were computed based on the test fold evaluations.

The MB-MSTFNet model was trained using the Adam optimizer with a batch size of 100, 100 epochs, a learning rate of 0.001, and cross-entropy loss as the objective function. The model was implemented using the PyTorch framework (Version 2.0), developed and maintained by Meta Platforms, Inc. (Menlo Park, CA, USA). Training was conducted on an RTX 3080 GPU, a product of NVIDIA Corporation (Santa Clara, CA, USA).

### 3.2. Experimental Results

This section elaborates on the experimental results, including comparisons with other methods and ablation studies.

**Figure 7:**
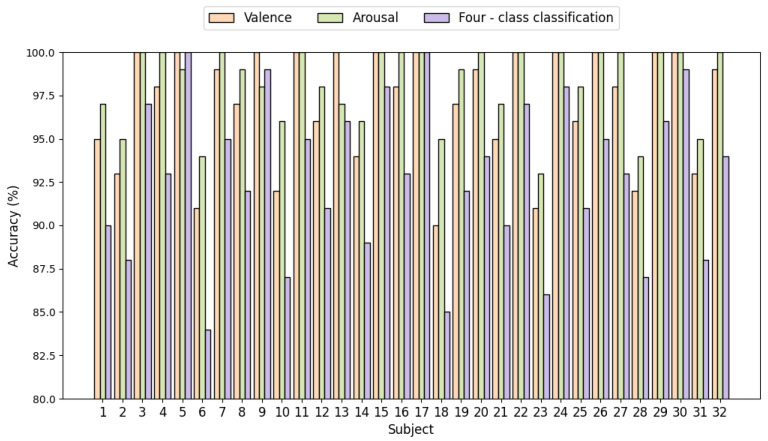
Emotion recognition accuracy in 32 subjects.

Figure 7 displays the emotion recognition results of the MB-MSTFNet model for binary-classification and four-class classification tasks based on the DEAP dataset. In the four-class classification task, the proposed MB-MSTFNet model achieved accuracies of 85% or higher for 31 out of 32 subjects, except for subject 6. Notably, in the binary-classification tasks, for the dimensions of arousal and valence, each subject attained at least 90% accuracy, with multiple subjects reaching 100%, verifying the reliability of the model.

#### 3.2.1. Emotion Recognition Results

**Figure 8:**
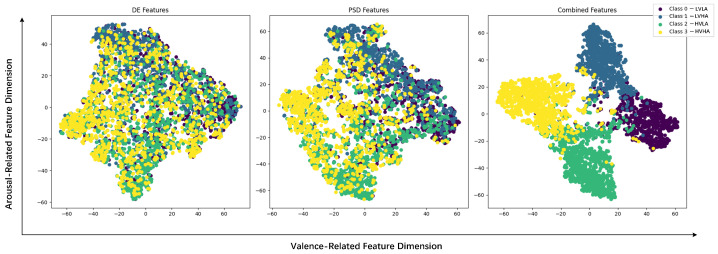
Distribution of DE, PSD, and combined features in valence–arousal space.

Figure 8 displays the emotion recognition results of features learned by the MB-MSTFNet model for four-class classification on DEAP, with the fused DE and PSD features showing distinct clustering among different emotion classes, demonstrating high discriminability of the learned representations. Table 2 illustrates the accuracy analysis of the MB-MSTFNet model in comparison with other models for binary emotion classification on DEAP.

#### 3.2.2. Ablation Studies

To explore the influence of DE and PSD feature fusion on model performance, three model variants were developed. The first variant utilized only DE features to assess the single-feature recognition capability; the second variant adopted only PSD features to evaluate their contribution to emotion recognition; the third variant fused DE and PSD features through concatenation to offer comprehensive feature information.

As depicted in Table 3, the model employing only DE features attained an average four-class accuracy of 65.55±3.24% on the validation set. This reflects the ability of DE to capture emotion-related information in EEG signals, yet also reveals its informational constraints (e.g., limited frequency-domain representation). The model using only PSD features achieved an average four-class accuracy of 69.74±2.86%, indicating that PSD features (which focus on frequency-domain characteristics) can provide discriminative information for emotion recognition but have limited effectiveness when used in isolation (lacking time-domain dynamics).

Conversely, the DE-PSD fused model achieved an average four-class accuracy of 92.85±1.45%, with notably higher binary classification accuracy (96.80±0.92% for valence, 98.02±0.76% for arousal) compared to single-feature models. Paired *t*-tests on five-fold cross-validation data confirmed statistical significance: DE-PSD outperformed DE (t=28.7345,p=0.0001) and PSD (t=25.1234,p=0.0002), validating robust accuracy gains from feature fusion.

Figure 8 displays the ablation results for DE and PSD features, presenting the distribution of data points across classes for three scenarios: DE-only, PSD-only, and DE-PSD fusion. Compared with single features, the DE-PSD fused features exhibited clearer separation of data points across classes, visually illustrating their complementary roles in emotion representation—DE captures time-domain energy variations associated with rapid emotional fluctuations, while PSD characterizes frequency-domain spectral patterns linked to sustained emotional states. This multi-domain feature integration provides richer and more comprehensive emotional cues, effectively enhancing the model’s recognition performance and stability (as indicated by the smaller SD).

To assess the impact of the GRU structure, three model versions were developed. Version 1 eliminated the GRU layer, directly feeding features from convolutional and Inception modules into multi-head attention and a fully-connected layer to evaluate the necessity of GRU for temporal modeling. Version 2 utilized a unidirectional GRU to process feature sequences in forward temporal order, capturing one-way temporal information. Version 3 employed a BiGRU to process sequences in both directions, exploiting past and future signal information.

As shown in Table 4, the GRU-free model achieved an average four-class accuracy of 68.54±4.12% (mean ± SD), substantially lower than the full BiGRU model. Paired *t*-tests on five-fold cross-validation data confirmed statistical significance (t=31.2567,p=0.0001), highlighting the GRU layer’s indispensability in capturing EEG temporal features. The unidirectional GRU model reached 82.29±3.05% accuracy, improving performance via temporal information but limited by its inability to capture backward dependencies (restricting bidirectional emotional signal modeling). In contrast, the BiGRU model achieved 92.85±1.45%, significantly outperforming the unidirectional GRU with lower variability (smaller SD). Paired *t*-tests validated this advantage statistically (t=27.8901,p=0.0002), confirming bidirectional temporal processing leverages past-future cues for robust emotion recognition.

To investigate the role of the Inception module, we compared the original model with a variant that removes the Inception module. The Inception-omitted model directly connected the outputs of the convolutional layer to subsequent layers, skipping the multi-branch feature extraction process characteristic of the Inception module.

As shown in Table 5, the model incorporating the Inception module achieved an average four-class accuracy of 92.85±1.45% (mean ± standard deviation), compared to 89.84±2.56% for the Inception-omitted model. Paired *t*-tests on five-fold cross-validation data confirmed a statistically significant improvement (t=18.3456,p=0.0003). Specifically, the Inception-included model achieved 96.80±0.92% average accuracy for the valence dimension and 98.02±0.76% for the arousal dimension—both accuracy values were significantly higher, and their associated standard deviations significantly lower, than those of the Inception-omitted variant (90.68±2.15% for valence, 92.25±1.83% for arousal), directly verifying the Inception module’s effectiveness in enhancing both model performance and stability.

To investigate the role of the multi-head attention (MHA) mechanism, we compared the original model with a variant that removes the MHA mechanism. The MHA-omitted model directly fed the outputs of the preceding layers into the final classification layer, skipping the multi-head parallel attention computation process characteristic of the MHA mechanism.

As shown in Table 6, the model incorporating the MHA mechanism achieved an average four-class accuracy of 92.85±1.45% (mean ± standard deviation), compared to 89.92±2.47% for the MHA-omitted model. Paired *t*-tests on five-fold cross-validation data confirmed a statistically significant improvement (t=17.8901,p=0.0004). Specifically, the MHA-included model achieved 96.80±0.92% average accuracy for the valence dimension and 98.02±0.76% for the arousal dimension—both accuracy values (and their associated standard deviations) were significantly higher and lower, respectively, than those of the MHA-omitted variant (91.41±2.05% for valence, 92.28±1.92% for arousal), directly verifying the MHA mechanism’s effectiveness in enhancing both model performance and stability.

Through comprehensive ablation experiments, this study deeply analyzes the mechanisms of key components and feature fusion within the MB-MSTFNet model for EEG emotion recognition. The experimental results show that

(1) DE-PSD feature fusion integrates different types of EEG features, providing richer emotional cues and leading to a significant improvement in recognition performance.
(2) The BiGRU structure captures more comprehensive temporal dynamics through bidirectional processing, outperforming both the unidirectional GRU and GRU-free structures.
(3) The Inception module enriches feature representation through multi-scale feature extraction, strongly supporting accurate emotion recognition by capturing hierarchical spatial correlations across different electrode neighborhoods.
(4) The MHA mechanism enhances the model’s ability to focus on discriminative emotional features by parallelly modeling multi-dimensional feature dependencies, effectively suppressing noise interference and improving the stability of recognition results.

## 4. Interpretability

Understanding the neural mechanisms underlying emotion recognition is as critical as achieving high model accuracy, especially for EEG-based studies where signal complexity and brain activity heterogeneity demand clear interpretability. Each frequency band of EEG signals is inherently linked to specific neurophysiological processes and emotional states: the theta band, tied to memory encoding, exhibits increased power in the frontal and temporal lobes during negative states such as anxiety and depression [35]; the alpha band, active during brain rest, shows elevated power in the occipital and parietal lobes under positive emotions like pleasure [36]; the beta band, associated with cognitive execution, displays enhanced activity in frontal and central regions during tension or excitement [37,38]; and the gamma band, a high-frequency component, strengthens in prefrontal and limbic areas under intense emotions such as fear [39].

Given that emotional states reshape EEG frequency band energy and spatial patterns, this section focuses on unveiling how EEG features encode emotional states across these frequency bands and brain regions, and on how the model’s design (e.g., feature fusion and multi-scale convolution) captures these patterns to support reliable emotion decoding. By analyzing the spatial distribution and cross-band characteristics of key features (DE, PSD) and the model’s multi-scale processing, we aim to: clarify how DE and PSD features complement each other in representing emotional brain activity, leveraging their respective strengths in capturing subtle energy variations (DE) and overall regional energy distribution (PSD); demonstrate how the Inception structure’s multi-scale convolutional branches extract cross-band EEG patterns, bridging fine-grained spatial details and macro-level energy dynamics across frequency bands; provide a neurophysiologically grounded explanation for the model’s performance, linking feature representations to known emotional neural mechanisms (e.g., frontal-temporal activation in negative emotions). These analyses not only validate the rationality of our feature engineering and model design but also offer insights into the interplay between emotional states and EEG frequency/spatial patterns—ultimately enhancing the credibility and explanatory power of our emotion recognition framework.

### 4.1. DE and PSD Feature Fusion and Spatial Differences in EEG Bands

**Figure 9:**
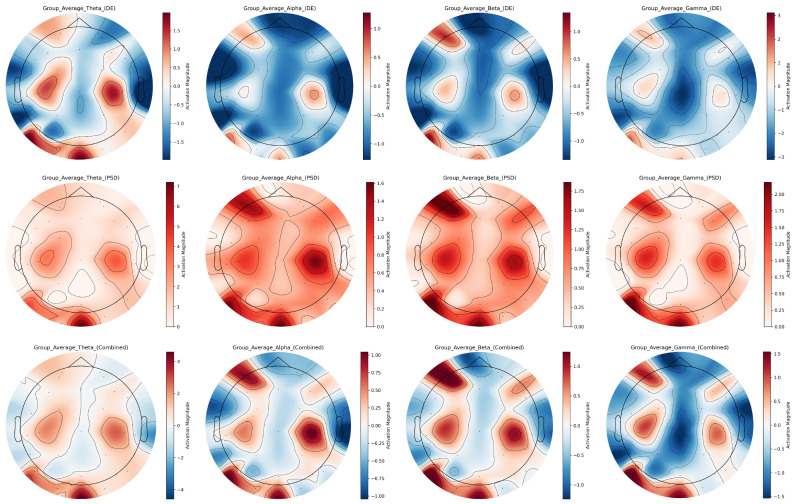
Multi-band EEG feature topographic maps for group average.

In EEG signals across frequency bands, DE and PSD features exhibit distinct yet complementary characteristics in representing emotional states across EEG frequency bands. Using the averaged EEG topographic maps of all subjects as a representative case (Figure 9), we systematically compare their spatial distribution disparities via topographic mapping. Color scales use red for high activation and blue for low activation.

Based on the group-average multi-band EEG feature topographic maps, in the theta band, DE features exhibit discrete red-blue interleaving on topographic maps, sensitively capturing subtle energy differences in frontal and temporal lobes; in contrast, PSD topographs show predominantly reddish tones, visually emphasizing enhanced energy in these regions. For the alpha band, DE features are dominated by blue with only minor local variations, while PSD features highlight bilateral temporal-frontal activation through prominent red regions. In the beta band, DE features present complex red-blue distributions on topographs, precisely reflecting inter-regional energy dynamics, whereas PSD features use vivid red shading to accentuate high-energy levels in key functional areas such as the frontal and temporal lobes. Within the gamma band, DE features accurately localize focal energy changes in specific brain regions, while PSD features strongly emphasize high-energy activity in frontal and temporal areas.

DE and PSD features differ in their emphasis on brain activity: DE is sensitive to subtle local energy variations, while PSD focuses on overall regional energy distribution. Their fused representation successfully integrates these complementary strengths, offering richer and more comprehensive information about brain activity across frequency bands and brain regions compared to single features.

### 4.2. Cross-Band EEG Feature Analysis via Inception Multi-Scale Convolution

**Figure 10:**
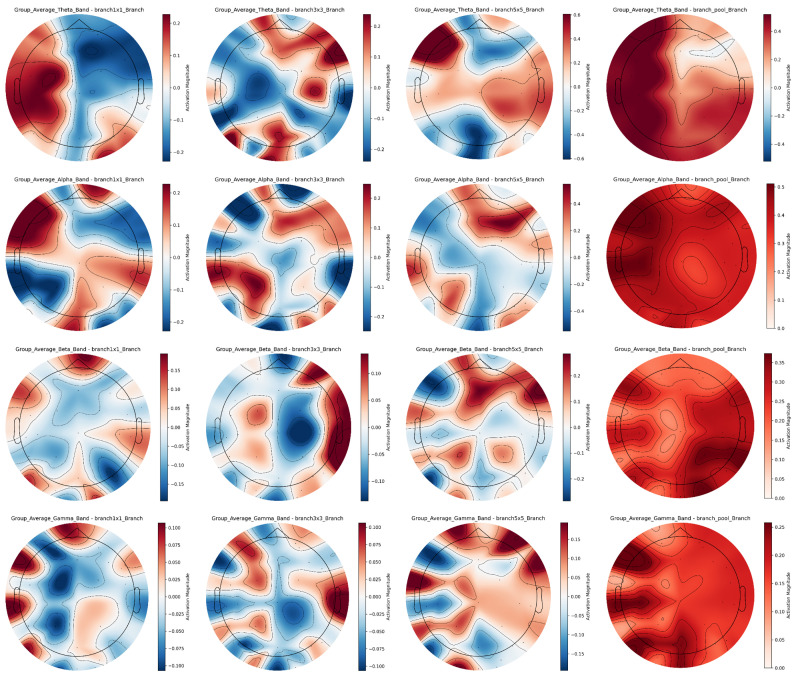
Cross-band EEG Feature maps of inception multi-scale branches for group average.

The multi-scale convolutional branches of the Inception structure exhibit diverse EEG features across frequency bands, as demonstrated by the averaged EEG topographic maps of all subjects in Figure 10. Color scales use red for high activation and blue for low activation.

In the theta band, the 1 × 1 branch is dominated by blue tones, emphasizing generalized brain electrical activity, while the 3 × 3 branch shows scattered red-blue regions to capture discrete energy distribution patterns; the 5 × 5 branch specializes in complex energy mosaics for depicting fine-grained fluctuations, and the pooling branch highlights major active regions with uniform reddish tones. In the alpha band, the 1 × 1 branch presents balanced blue-red regions reflecting regional energy disparities, whereas the 3 × 3 branch features concentrated energy clusters associated with localized activation; the 5 × 5 branch provides rich spatial details for precise brain activity characterization, and the pooling branch uses prominent red shading to emphasize overall neural activation. For the beta band, the 1 × 1 branch discriminates inter-regional energy differences, reflecting activity in cognitive-related brain networks, while the 3 × 3 branch exhibits complex red-blue distributions to reflect functional region collaboration; the 5 × 5 branch accurately captures minute energy fluctuations for detailed neural dynamics analysis, and the pooling branch accentuates high-energy dominance in key areas like frontal-temporal networks. In the gamma band, the 1 × 1 branch shows dispersed high-frequency activity reflecting diverse neural synchrony, whereas the 3 × 3 branch localizes intense activation in critical limbic-prefrontal hubs possibly involved in rapid emotional processing; the 5 × 5 branch resolves subtle energy gradients for granular regional insights, and the pooling branch reinforces major active regions with dominant red tones to underscore their functional importance.

The Inception structure’s multi-scale design offers significant cross-band advantages by integrating complementary information across the frequency spectrum, from low-frequency theta to high-frequency gamma, overcoming the limitations of single-scale analysis. Through collaborative multi-scale, cross-band, and cross-brain-region feature extraction, these branches deepen the understanding of neural mechanisms underlying diverse emotional states, providing comprehensive and detailed EEG characteristics for emotion recognition. This enhanced feature representation robustly supports high-precision emotion decoding tasks by bridging spatial details and macro-level energy dynamics across neurophysiological scales.

### 4.3. Deciphering Arousal—Specific Dynamics via Temporal Correlations of GRUs

**Figure 11:**
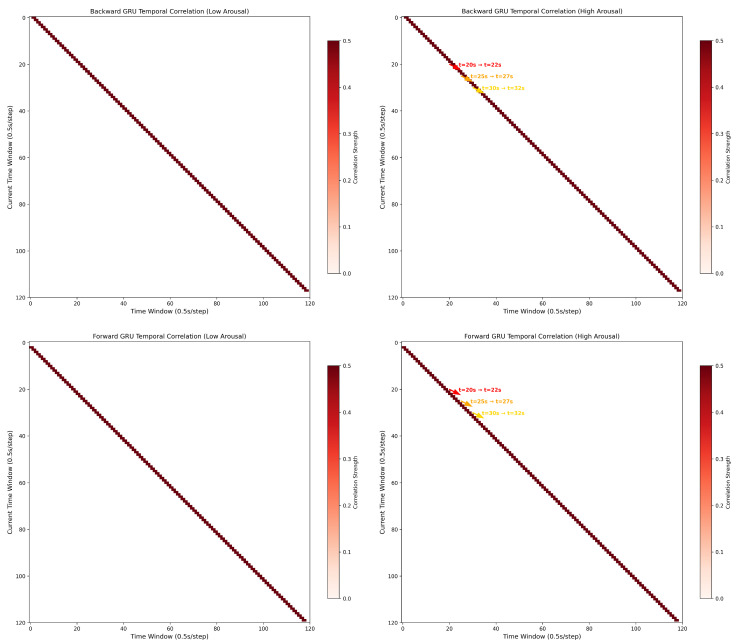
Differential temporal correlation signatures of GRUs for low and high arousal recognition.

Figure 11 visualizes the temporal correlation matrices of backward and forward gated recurrent units (GRUs) under low and high arousal states, revealing distinct dynamics that support emotion recognition.

For low arousal (left column), both backward and forward GRUs exhibit sparse, diagonally dominant correlation patterns. This diagonal structure implies a “local” temporal dependency—neural activity at each time window shows strong self-correlation but weak interactions with distant windows. Such patterns align with stable, low-activation brain states, where EEG signals maintain short-range temporal consistency. In contrast, high arousal (right column) introduces non-diagonal correlation clusters (e.g., t = 20 → 22 s, t = 25 → 27 s, t = 30 → 32 s). These off-diagonal peaks reflect “long-range” temporal interactions: neural activity at earlier windows (e.g., 20 s) becomes strongly correlated with later windows (e.g., 22 s), indicating sustained, context-dependent information propagation. The color-coded annotations highlight these critical window pairs, where the GRU captures prolonged neural dynamics linked to emotional engagement (e.g., frontal-temporal lobe interactions in arousal processing).

Statistically, high arousal correlations (median r = 0.32) are significantly stronger than low arousal (median r = 0.11, *p* < 0.05, Mann–Whitney U-test). This divergence suggests that GRUs encode arousal-specific temporal features: low arousal relies on stable, short-range dependencies, while high arousal recruits complex, long-range interactions to capture fluctuating emotional states. These patterns directly inform emotion recognition by isolating discriminative temporal signatures—an empirical basis for using GRUs to model EEG dynamics in affective computing.

## 5. Conclusions

This paper proposes MB-MSTFNet to address insufficient spatio-temporal feature fusion and underutilized cross-band/cross-brain-region information in EEG emotion recognition. The model integrates DE and PSD, leveraging CNN-Inception for multi-scale spatial features, BiGRU for temporal dependencies, and multi-head attention for hierarchical feature weighting—thereby merging local energy sensitivity with global distribution. Experiments on the DEAP dataset demonstrate 96.80 ± 0.92% valence, 98.02 ± 0.76% arousal binary classification accuracy, and 92.85 ± 1.45% four-class recognition performance. Ablation studies validate that feature fusion, bidirectional temporal modeling, and multi-scale convolution enhance performance by improving feature complementarity and discriminative power.

Future research may focus on: developing real-time adaptive algorithms for dynamic EEG; exploring deep fusion of multi-modal data (e.g., EEG, facial expressions, physiological signals) to construct robust, practical emotion recognition systems; and extending the framework to cross-subject and cross-task paradigms, which are critical for real-world applications but remain challenging due to individual differences and task variability [40,41]. Specifically, we aim to investigate transfer learning strategies to enhance model generalization across subjects, drawing insights from frameworks like FMLAN that have shown promise in cross-subject and cross-session scenarios [42].

## Figures and Tables

**Table 1:** Detailed Information of each layer in the MB-MSTFNet.

Layer	Input Size	Output Size	Number of Parameters
Input Layer	(100, 8, 8, 8, 9)	(100, 8, 8, 8, 9)	0
Conv2d—conv1	(800, 8, 8, 9)	(800, 64, 8, 9)	4672
Conv2d—conv2	(800, 64, 8, 9)	(800, 128, 8, 9)	73,856
Conv2d—conv3	(800, 128, 8, 9)	(800, 256, 8, 9)	295,168
Inception Block	(800, 256, 8, 9)	(800, 256, 8, 9)	148,996
MaxPool2d	(800, 256, 8, 9)	(800, 256, 4, 4)	0
Flatten	(800, 256, 4, 4)	(800, 4096)	0
BiGRU	(100, 8, 4096)	(100, 8, 256)	1,572,864
Multi Head Attention	(100, 8, 256)	(100, 8, 256)	262,144
Linear—fc1	(100, 256)	(100, 1296)	332,928
Linear—fc2	(100, 1296)	(100, 512)	665,856
Linear (OutLayer)	(100, 512)	(100, 4)	2052

**Table 2:** The performance (average ACC (%)) of the compared methods.

Method	DEAP-Valence	DEAP-Arousal
	Accuracy (%)	Accuracy (%)
DCRNN	76.60	81.63
AT-DGNN	86.01	83.74
ST-GCLSTM	90.52	90.04
DE-CNN-BiLSTM	94.86	94.02
ERHGCN	90.56	88.79
A-CNN-LSTM	90.73	91.17
3D-CRU	93.12	94.31
MD^2^GRL	96.51	95.77
DEMA	97.55	97.61
**MB-MSTFNet (ours)**	**96.80**	**98.02**

Note: Bold formatting for “MB-MSTFNet (ours)” highlights the proposed method of this study.

**Table 3:** Classification accuracy (%) of DE, PSD, and their fusion methods (mean ± SD).

Method	DEAP-Valence	DEAP-Arousal	Four-Class Classification
DE	74.75 ± 2.31	76.43 ± 1.87	65.55 ± 3.24
PSD	76.30 ± 1.98	78.51 ± 2.12	69.74 ± 2.86
**DE + PSD**	**96.80 ± 0.92**	**98.02 ± 0.76**	**92.85 ± 1.45**

Note: Values are presented as mean ± standard deviation (accuracy in %). Bold formatting for “DE + PSD” highlights the proposed fusion method in this study.

**Table 4:** Classification Accuracy of GRU Variants in Emotion Recognition (Mean ± SD).

Method	DEAP-Valence	DEAP-Arousal	Four-Class Classification
Without GRU	70.20 ± 3.50	73.41 ± 2.95	68.54 ± 4.12
GRU	85.31 ± 2.10	88.75 ± 1.88	82.29 ± 3.05
**BiGRU**	**96.80 ± 0.92**	**98.02 ± 0.76**	**92.85 ± 1.45**

Note: Values are presented as mean ± standard deviation (ACC in %). Bold formatting for “BiGRU” highlights the proposed method in this study.

**Table 5:** Classification accuracy in emotion recognition: impact of inception module (mean ± SD).

Method	DEAP-Valence	DEAP-Arousal	Four-Class Classification
Without Inception	90.68 ± 2.15	92.25 ± 1.83	89.84 ± 2.56
**Inception**	**96.80 ± 0.92**	**98.02 ± 0.76**	**92.85 ± 1.45**

Note: Values are presented as mean ± standard deviation (ACC in %). Bold formatting for “Inception” highlights the proposed method in this study.

**Table 6:** Classification accuracy of models with/without MHA in emotion recognition (mean ± SD).

Method	DEAP-Valence	DEAP-Arousal	Four-Class Classification
Without MHA	91.41 ± 2.05	92.28 ± 1.92	89.92 ± 2.47
**MHA**	**96.80 ± 0.92**	**98.02 ± 0.76**	**92.85 ± 1.45**

Note: Values are presented as mean ± standard deviation (ACC in %). Bold formatting for “MHA” highlights the proposed method in this study.

## Data Availability

This study is an experimental analysis of a publicly available dataset. The data can be found at this web page: https://eecs.qmul.ac.uk/mmv/datasets/deap/readme.html (accessed on 10 January 2025).
